# Exploring the prognostic differences in patients of Chiari malformation type I with syringomyelia undergoing different surgical methods

**DOI:** 10.3389/fneur.2022.1062239

**Published:** 2023-01-04

**Authors:** Mingchu Zhang, Yan Hu, Dengpan Song, Chengcheng Duan, Mingkun Wei, Longxiao Zhang, Shixiong Lei, Fuyou Guo

**Affiliations:** ^1^Department of Neurosurgery, The First Affiliated Hospital of Zhengzhou University, Zhengzhou, Henan, China; ^2^International Joint Laboratory of Chiari Malformation, Zhengzhou, Henan, China; ^3^Department of Neurosurgery, Beijing Hospital, Beijing, China

**Keywords:** Chiari malformation type I, syringomyelia, posterior fossa decompression with resection of tonsils (PFDRT), posterior fossa decompression with duraplasty (PFDD), prognostic factors

## Abstract

**Background:**

The best surgical treatment of Chiari malformation patients with syringomyelia remains controversial, and whether cerebellar tonsillectomy should be performed has not been decided.

**Objective:**

To evaluate the efficacy of posterior fossa decompression with duraplasty (PFDD) and Posterior fossa decompression with resection of tonsils (PFDRT) in patients of Chiari malformation type I (CM-I) with syringomyelia and explore relevant factors affecting prognosis.

**Patients and methods:**

We retrospectively analyzed 182 adult patients of CM-I with syringomyelia who underwent PFDD or PFDRT over a 6-year period, and analyzed their clinical manifestations, imaging features, and follow-up data. Clinical outcomes were assessed using the Chicago Chiari Outcome Scale (CCOS), and imaging outcomes were assessed using the syrinx remission rate. Difference comparisons were performed to compare the differences between different surgical groups. Influencing factors associated with outcome were investigated using bivariate analysis and multiple linear regression analysis.

**Results:**

There were statistically significant differences in CCOS score (*p* = 0.034) and syrinx remission rates (*p* = 0.046) between the PFDRT group and the PFDD group after surgery. Regression analysis showed that preoperative motor dysfunction, cerebellar-related symptoms and different surgical methods may have influenced the CCOS score and that brainstem-related symptoms and age may have influenced the syrinx remission rates in the total patient group (*p* < 0.05). Regression analysis showed that the duration of symptoms, cerebellar-related symptoms and preoperative syrinx diameter may have influenced the CCOS score and that the preoperative cerebellar tonsillar hernia distance may have influenced the postoperative syrinx remission rate in the PFDRT group (*p* < 0.05). Age and length of hospital stay may have influenced the CCOS score, and brainstem-related symptoms and age may have influenced the syrinx remission rates in the PFDD group (*p* < 0.05).

**Conclusion:**

This study showed that the CCOS score in the PFDRT group was better than that in the PFDD group. Preoperative motor dysfunction, cerebellar-related symptoms, and different surgical methods in patients of CM-I with syringomyelia affected postoperative CCOS score. Both the duration of symptoms and the age of the patients should be actively considered as factors influencing prognosis. Symptomatic CM-I patients with syringomyelia should undergo surgical treatment as early as possible.

## 1. Introduction

Chiari malformation type I (CM-I) is a relatively rare congenital disease that is thought to be caused by posterior fossa (PCF) dysplasia resulting from paraxial mesodermal disease (1). The prevalence of CM-I in adults ranges from ~0.24 to 0.9% (2–4). Chiari I malformation is defined radiographically as an inferior displacement of the one or both cerebellar tonsils of 5 mm below the opisthion-basion line (5–7). Tonsil descent < 3 mm is considered a physiological variation (normal MRI), while between 3 and 5 mm a borderline ectopia, deserving observational approach in symptomatic cases, especially in the presence of syrinx or peg-like tonsillar profile (5). The most common cause of syringomyelia is CM-I, which accounts for ~40.0–80.5% of cases (8). The progression of syringomyelia associated with Chiari I is produced by the action of the cerebellar tonsils. The ectopic cerebellar tonsils descended into the cervical spinal canal during the cardiac systole, acting on the enclosed spinal subarachnoid space, creating enlarged spinal subarachnoid pressure waves, and driving cerebrospinal fluid from the subarachnoid space into the perivascular and extracellular space of the spinal cord, resulting in syrinx formation (9–11).

The presence of syringomyelia is an indication for surgery in patients with CM-I (5). In current studies, suboccipital decompression surgery is the preferred treatment option for CM-I patients, but there is no general consensus on the preferred, specific decompression method (12, 13). Posterior fossa decompression with duraplasty (PFDD) is a commonly performed surgical modality and has been proven to be an effective first-line treatment strategy (3, 5, 14). However, With the further in-depth study of CM-I, posterior fossa decompression with resection of tonsils (PFDRT) is considered to further resolve the subarachnoid cerebrospinal fluid flow obstruction in the area of the occipital bone, increase the volume of the occipital pool, and improve the cerebrospinal fluid circulation at the craniovertebral junction (15). However, many neurosurgeons debate whether to perform PFDRT because of its invasiveness and lack of high-quality evidence (16). To further evaluate the efficacy of PFDD and PFDRT in patients of CM-I with syringomyelia and explore relevant factors affecting the prognosis, we conducted a retrospective analysis of 182 patients who met the criteria in our institution. Imaging changes were assessed by using the syringomyelia remission rate, and clinical improvement was assessed by using the CCOS score. To the best of our knowledge, this study is one of the largest single-center clinical studies currently available.

## 2. Materials and methods

### 2.1. Patient population

The cohort included 182 patients of CM-I with syringomyelia who were admitted to the neurosurgery department at our institution between January 2016 and December 2021. All participants signed the consent form approved by the Hospital Ethics Committee and the Institutional Research Committee. We collected basic information about the patients, including age, sex, blood pressure reading, duration of symptoms, operation time, length of hospital stay, and imaging characteristics. We divided the patients into 2 groups according to surgical procedure, with 101 patients in the PFDD group and 81 patients in the PFDRT group. Inclusion criteria: (1) 3.0 T magnetic resonance imaging (MRI) of the craniocervical junction region showing a cerebellar tonsil hernia larger than 5 mm before surgery (2) CM-I combined with syringomyelia (3) Age> 18 years. (4) Complete clinical data. (5) Sufficient follow-up time (≥6 months). Exclusion criteria: (1) Secondary syringomyelia caused by spinal cord tumor or spinal injury. (2) Other diseases leading to cerebellar subtonsillar hernia, such as Hydrocephalus, Idiopathic Intracranial Hypertension (6) (3) Other types of CM with or without syringomyelia. (4) Treated for CM-I combined with syringomyelia with other surgical modalities. (5) History of posterior fossa surgery. (6) Cranial and neck connection instability.

### 2.2. Clinical and imaging characteristics

We classified the clinical symptoms of the patients into the following categories: (1) Paraesthesia, including numbness and pain (2) cough headache (3) Noncough headache (4) Motor dysfunction, including weakness of the limbs and muscle atrophy (5) brainstem-related symptoms: dysphagia and hoarseness (6) sensory deficits, including pain and warm sensory separation (7) cerebellar-related symptoms, including ataxia and gait instability (8) Other neurological disorders, including diplopia, tinnitus, and bladder incontinence.

**Figure 1:**
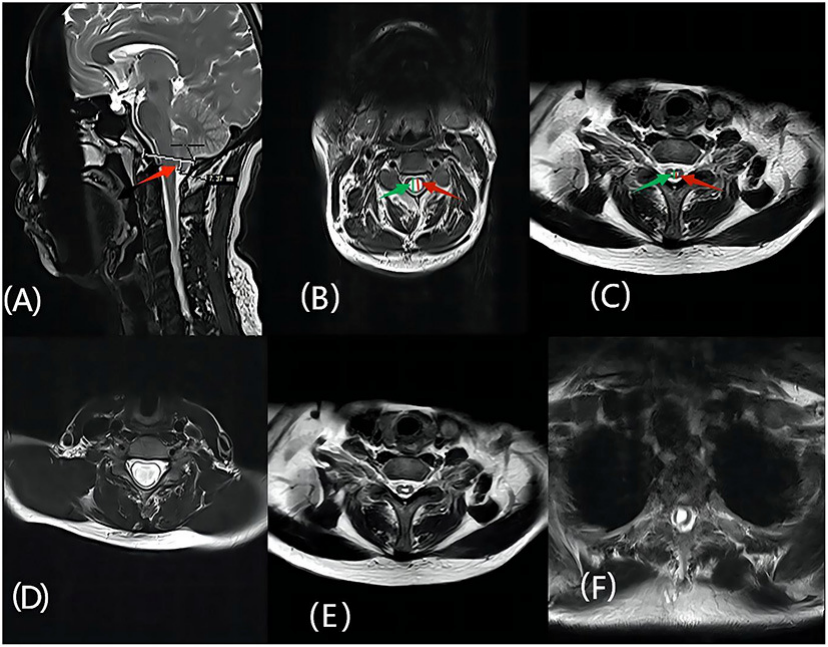
**(A)** The red arrow represents the herniated cerebellar tonsil; **(B)** The green arrow represents the diameter of the patient's spinal cord before surgery, defined as b, the red arrow represents the maximum anteroposterior diameter of the preoperative syrinx, which is defined as a; **(C)** The red arrow represents the maximum anteroposterior diameter of the postoperative syrinx, defined as a1, the green arrow indicates the maximum diameter of the spinal cord after surgery, defined as b1; **(D–F)** represent the morphological features of the syringomyelia, which are enlarged, central, and deviated, respectively.

All patients underwent MR scans of the brain and the entire spinal cord using the 3.0 T MR imaging system (Siemens). Based on the MR imaging data of the patient examined in our hospital before and after surgery, we selected the median sagittal position to measure the cerebellar subtonsillar hernia distance and the characteristics of the syrinx (location, and type; i.e., central, enlarged, or deviated) (17). The axis was selected to measure the relevant data. The preoperative maximum diameter of the syrinx was defined as a, and the spinal cord diameter as b. The postoperative maximum diameter of the syrinx was defined as a1, and the postoperative spinal cord diameter was defined as b1. The preoperative spinal cord ratio was a/b, and the postoperative spinal cord ratio was a1/b1. These measurements were independently assessed by two neurosurgical researchers at our institution and averaged (Figure 1).

### 2.3. Surgical procedure

All patients were operated on in the prone position with the patient's head fixed with a three-pin holder, and the neck slightly flexed to obtain an open angle between the occipital bone and upper cervical laminae. The occipital bone and the posterior arch of the C1 vertebra were exposed in all cases, and the lamina of the C2 vertebra was exposed if tonsillar herniation extended to this level. Both groups underwent 2.5^*^3 cm occipital caliectomy with a C1 posterior vertebral arch and C2 and C3 laminectomy if necessary. PFDD group: The dura was incised in a Y-shape under the microscope and held open by sutures. Then, we explored fiber membrane formation in the Magendie foramen; if present, arachnoid adhesions were released by sharp dissection to facilitate the CSF flow. The operative region was rinsed with dexamethasone solution to reduce blood infiltration and adhesions. Waterproof closure of the duraplasty with non-resorbable stitches and autograft was used to prevent CSF leakage and to reconstruct a Cisterna Magna. Finally, the incision was meticulously closed in anatomical layers (5, 18).

PFDRT group: the dura was opened using the same method. We loosened the arachnoid adhesion among the cerebellar tonsils, medulla oblongata and spinal cord. To resect the herniated cerebellar tonsils while protecting the brainstem, we placed a gelatin sponge between the cerebellar tonsils and the brainstem. Cerebellar tonsillectomy was then performed by subpial resection. Subpial operation is necessary for this step in order to protect the surrounding structures, such as arteriae cerebelli inferior posterior (18). In the same way, we explored fiber membrane formation in the Magendie foramen to fully open up the cerebrospinal fluid circulation. Using the same method as in the PFDD group, we treated the operative area and the dura. To avoid differences in surgical procedures causing different outcomes as much as possible, all procedures were performed by experienced surgeons at our institution.

### 2.4. Outcome assessment

All patients underwent MRI 6 months after surgery. Patients who come to our hospital regularly will be followed up by MRI review combined with CCOS score, and others will receive regular telephone follow-up. In this study, we analyzed the results according to the patients' last follow-up. Radiological outcome was measured as the percentage of regression of syringomyelia on MRI using the following formula: [(preoperative syrinx/cord ratio – follow-up syrinx/cord ratio) × 100]/(preoperative syrinx/cord ratio) (19). Clinical improvement was assessed by using the CCOS. Many studies have shown that the CCOS system is considered the only CM-I-specific outcome scale associated with widely accepted outcome measures (20–22). This scale includes 4 outcome categories (pain, non-pain symptoms, functionality, and complications) that are scored from 1 to 4; the maximal total score is 16, and the higher the score, the better the prognosis (21).

### 2.5. Statistical analysis

Parametric data are expressed as the mean ± SD and were compared using a *t-*test. Nonparametric data were expressed as medians and compared using McNemar's test (only the duration of symptoms was nonparametric data), and frequency distributions were used to describe the categorical variables. Hypothesis testing was performed at each stage to remove outliers, assess normality, and check correlations. The Anderson normality test was used to assess the normality of the distribution of the variables. Dixon's Q test was used for the identification and rejection of outliers. Difference comparisons (using the independent sample *t-*test, the chi-square test, Fisher's exact test) were performed to compare the differences between the different surgical groups. Bivariate analysis (using the chi-square test, Pearson's correlation, Point biserial correlation test) was performed to identify the preoperative variables that correlated with the outcome measures. Multiple linear regression analysis was then performed using the variables with a *p*-value < 0.05 on the bivariate analysis to check for their independent associations with the outcome measures.

## 3. Results

### 3.1. Preoperative information

There were 52 (28.4%) male and 130 female (71.6%) patients in the study cohort, with a mean ± SD age of 46.39 ± 10.58 years (range 18–74 years). The disease duration ranged from 1 to 290 months. The main clinical manifestations of the patients were divided into the following: (1) paraesthesia, 92 (50.5%); (2) cough headache, 62 (34.1%); (3) noncough headache, 15 (8.2%); (4) motor dysfunction, 53 (29.1%); (5) brainstem-related symptoms, 4 (2.1%); (6) sensory deficits, 29 (15.9%); (7) cerebellar-related symptoms, 12 (6.5%); and (8) other neurological disorders, 7 (3.8%). The syrinx was observed in the MRI sagittal position in 54 (29.6%) patients with a cervical syrinx, 126 (69.2%) with a cervicothoracic syrinx and 2 (1.2%) with a holocord syrinx. The distance of tonsillar herniation ranged from 5.0 to 26.3 mm (mean 8.88 ± 4.09 mm). The preoperative syrinx/cord ratio was 0.63 ± 0.22. In regard to the preoperative information, only paresthesia was significantly different between the two surgical groups (*p* = 0.032, < 0.05). A detailed summary is shown in Tables 1, 2.

**Table 1:** Demographic and clinicoradiological characteristics before operation.

**Feature**	**Total**	**PFDRT (*n =* 81)**	**PFDD (*n =* 101)**	**Value**
**Sex**				
Male	52 (28.4%)	21 (25.9%)	31 (30.6%)	0.479
Female	130 (71.6%)	60 (74.1)	70 (69.4%)	
Age mean ± SD (years)	46.39 ± 10.58	46.46 ± 10.90	46.34 ± 10.30	0.940
Duration of symptoms (months)	24 (6, 96)	12 (4, 84)	24 (6, 96)	0.309
Time of surgery (hours)	3.19 ± 1.00	3.14 ± 1.07	3.24 ± 0.95	0.527
Time of hospital stay (days)	19.66 ± 7.43	19.84 ± 7.79	19.52 ± 7.12	0.778
**Blood pressure**				
No hypertension	123 (67.5%)	54 (66.7%)	69 (68.3%)	0.874
Hypertension	59 (32.5%)	27 (33.3%)	32 (31.7%)	
Paraesthesia	92 (50.5%)	48 (59.3%)	44 (43.6%)	0.032^*^
Cough headache	62 (34.1%)	28 (34.6%)	34 (33.7%)	0.898
Non-cough headache	15 (8.2%)	9 (11.1%)	6 (5.9%)	0.207
Motor dysfunction	53 (29.1%)	21 (25.9%)	32 (31.7%)	0.396
Brainstem-related symptoms	4 (2.1%)	2 (2.5%)	2 (2.0%)	1.000
Other neurological disorders	7 (3.8%)	3 (3.87%)	4 (4.0%)	1.000
Cerebellar-related symptoms	12 (6.5%)	7 (8.6%)	5 (5.0%)	0.319
Sensory deficits	29 (15.9%)	10 (12.3%)	19 (18.8%)	0.236
**Syrinx location**, ***n*** **(%)**				
Holocord	2 (1.2%)	1 (1.2%)	1 (0.9%)	0.117
Cervicothoracic	126 (69.2%)	52 (64.1%)	74 (73.2%)	0.372
Cervical	54 (29.6%)	28 (34.5%)	26 (25.7%)	0.197
The maximum anteroposterior diameter of syrinx before surgery (mm)	5.72 ± 2.99	5.82 ± 2.97	5.64 ± 3.01	0.700
Syrinx/cord ratio before surgery	0.63 ± 0.22	0.63 ± 0.22	0.64 ± 0.23	0.808
Tonsillar herniation before surgery (mm)	8.88 ± 4.09	8.48 ± 3.60	9.25 ± 4.42	0.211

Total means total patients group; ^*^p < 0.05.

**Table 2:** Clinicoradiological characteristics after operation.

**Feature**	**Total**	**PFDRT (*n =* 81)**	**PFDD (*n =* 101)**	**Value**
Syrinx/cord ratio after surgery	0.38 ± 0.21	0.35 ± 0.20	0.42 ± 0.22	0.034^*^
syrinx remission rate	0.40 ± 0.29	0.44 ± 0.28	0.35 ± 0.31	0.046^*^
Tonsillar herniation after surgery (mm)	2.52 ± 3.35	2.33 ± 3.09	2.67 ± 3.57	0.500
The cerebellar tonsils above the occipital foramen after surgery	42 (23.1%)	26 (32.1%)	16 (15.8%)	0.010^*^
CCOS Score	13.45 ± 1.87	13.78 ± 1.67	13.19 ± 1.98	0.034^*^
Postoperative aseptic inflammation	21 (11.5%)	13 (16.0%)	8 (7.9%)	0.088
Subcutaneous effusion	10 (5.5%)	5 (6.2%)	5 (5.0%)	0.974
Pseudomeningocele	3 (1.6%)	2 (2.5%)	1 (1.0%)	0.847
Secondary surgery	3 (1.6%)	2 (2.5%)	1 (1.0%)	0.847
Syrinx resolution	13 (7.1%)	8 (9.9%)	5 (4.9%)	0.649
Syrinx remained the same or worse	29 (15.9%)	12 (14.8%)	17 (16.8%)	0.712

Total means total patients group; ^*^p < 0.05.

### 3.2. Postoperative follow-up

**Figure 2:**
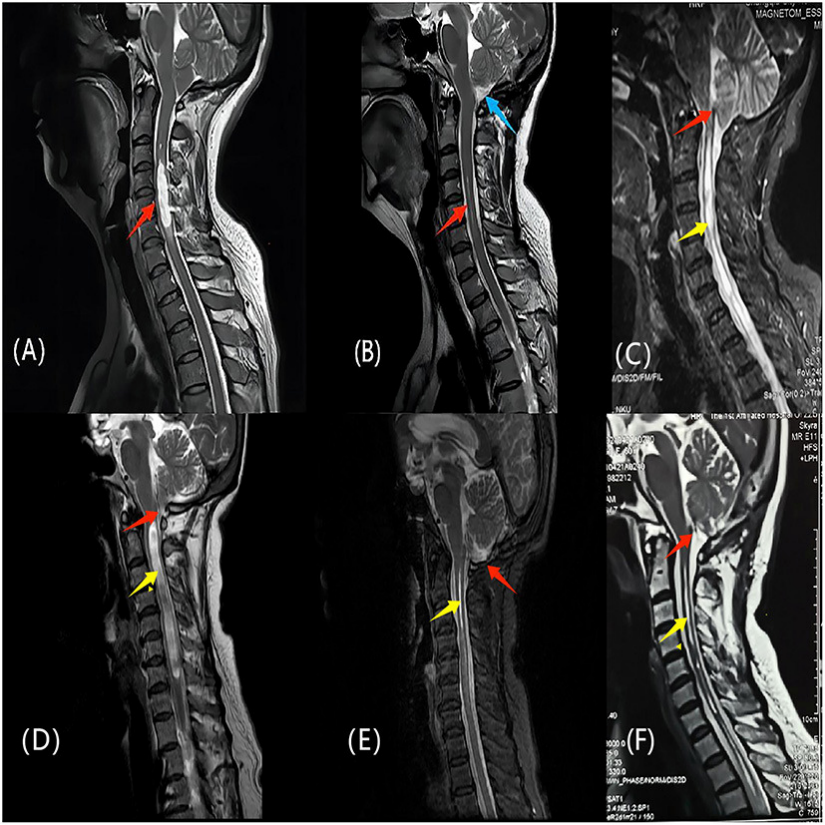
**(A, B)** One patient of CM-Iwith syringomyelia underwent PFDRT. **(A)** The red arrow indicates the syringomyelia before operation; **(B)** The syrinx indicated by the red arrow almost disappeared, and the blue arrow indicates the indirect increase of the cisterna magna after the removal of the cerebellar tonsils. **(C, F)** another patient of CM-Iwith syringomyelia underwent PFDRT. **(C)** The red arrow indicates the obstructive state in the Magendie foramen; **(F)** The red arrow indicates that restricted CSF circulation in the Magendie foramen was improved after operation. The yellow arrows indicate a significant improvement of the syringomyelia. **(D, E)** The patient of CM-Iwith syringomyelia was treated with PFDD. Red arrows indicates the cerebellar tonsils and the yellow arrows indicates the syringomyelia. **(E)** Two years later after operation, the cerebellar tonsils returned to be normal and the syrinx almost disappeared. The clinical symptoms of the above three patients were significantly improved at follow-up.

Follow-up data were available for all patients, with a mean follow-up time of 24 months. The mean follow-up period of the PFDRT group was 22.23 ± 15.78 months, and that of the PFDD group was 23.52 ± 17.03 months. According to the follow-up results, the mean postoperative CCOS score of the patients in the total population group was 13.45 ± 1.87, and the CCOS scores in the PFDRT group were 13.78 ± 1.67 and in the PFDD group were 13.19 ± 1.98 (*p* = 0.034). Postoperative MRI showed the loss of the syrinx in 8 (9.9%) and 5 (4.9%) patients in the PFDRT and PFDD groups, respectively (Figure 2). There were 12 (14.8%) and 17 (16.8%) patients whose syringomyelia was aggravated or unchanged in the PFDRT and PFDD groups, respectively. There were statistically significant differences in the syrinx remission rates between the PFDRT group and the PFDD group after surgery (*p* = 0.046). A detailed summary is shown in Table 1. Patients who suffered from motor dysfunction had the lowest improvement rate, which was 66.03%. However, patients who suffered from cough headache had the best improvement rate, which was up to 93.55%. Detailed results are shown in Supplementary Table 1.

### 3.3. Data analysis on the CCOS score

The duration of symptoms, motor dysfunction, cerebellar-related symptoms, and different surgical methods in the total patient group were correlated with postoperative CCOS score (*p* < 0.05). After the related variables were included in the multiple linear regression analysis, it was found that motor dysfunction, cerebellar-related symptoms, and different surgical methods were independent influencing factors of postoperative CCOS score (*p* < 0.05). Motor dysfunction and cerebellar-related symptoms were negatively correlated with postoperative CCOS scores. The scores were higher in the PFDRT group than in the PFDD group.

Bivariate correlation analysis showed that the duration of symptoms, cerebellar-related symptoms, preoperative syrinx diameter, and preoperative syrinx/cord ratio CCOS scores were correlated with postoperative CCOS score in the PFDRT group (*p* < 0.05). Relevant variables were included in the multiple linear regression analysis, and the duration of symptoms, cerebellar-related symptoms, and preoperative syrinx diameter in the PFDRT group affected CCOS scores.

In the PFDD group, age, length of stay, and motor dysfunction were significantly different from the CCOS scores. Multiple linear regression analysis showed that patient age and length of hospital stay were independent risk factors for low postoperative CCOS scores in the PFDD group. The above detailed results are shown in Tables 3, 4.

**Table 3:** CCOS bivariate correlation analysis.

**Parameters**	**Total**		**PFDRT**		**PFDD**	
	***r***	***p***	***r***	***p***	***r***	***p***
Sex	−0.078	0.295	−0.093	0.408	−0.049	0.624
Age	−0.140	0.060	−0.017	0.881	−0.233	0.019^*^
Duration of symptoms	−0.248	0.001^*^	−0.330	0.003^*^	−0.147	0.143
Time of surgery	0.055	0.457	0.126	0.264	0.018	0.859
Time of hospital stay	−0.107	0.149	0.159	0.156	−0.314	0.001^*^
Blood Pressure	−0.025	0.741	0.079	0.485	−0.126	0.211
Paraesthesia	−0.034	0.652	0.040	0.724	−0.139	0.166
Cough headache	0.119	0.110	0.094	0.405	0.143	0.154
Non-cough headache	0.087	0.244	0.084	0.457	0.065	0.518
Motor dysfunction	−0.161	0.030^*^	−0.086	0.443	−0.210	0.035^*^
Brainstem–related symptoms	−0.020	0.786	−0.050	0.656	0.001	0.990
Other neurological disorders	−0.136	0.067	−0.166	0.138	−0.114	0.255
Cerebellar–related symptoms	−0.246	0.001^*^	−0.418	0.000^*^	−0.084	0.406
Sensory deficits	−0.145	0.051	−0.073	0.515	−0.186	0.062
Syrinx location	0.035	0.640	0.024	0.832	0.023	0.820
The maximum anteroposterior diameter of syrinx before surgery	−0.103	0.166	−0.346	0.002^*^	0.048	0.632
Syrinx/cord ratio before surgery	−0.056	0.452	−0.219	0.049^*^	0.051	0.610
Tonsillar herniation before surgery	−0.024	0.752	−0.082	0.466	0.031	0.757
Surgical method	−0.139	0.041^*^			

Total means total patients group; ^*^p < 0.05.

**Table 4:** CCOS multiple regression analysis.

**Variable**	**Beta**	**SE**	***P*-value**
**Total**			
Constant	14.87	0.441	0
Duration of symptoms	−0.003	0.002	0.103
Motor dysfunction	−0.731	0.289	0.012^*^
Cerebellar-related symptoms	−1.915	0.531	0.000^*^
Surgical method (PFDRT)	0.585	0.264	0.028^*^
**PFDRT**			
Constant	14.996	0.521	0
The maximum anteroposterior diameter of syrinx before surgery	−0.419	0.116	0.001^*^
Duration of symptoms	−0.007	0.002	0.006^*^
Syrinx/cord ratio before surgery	2.79	1.263	0.09
Cerebellar-related symptoms	−2.532	0.539	0.000^*^
**PFDD**			
Constant	17.372	1.011	0
Age	−0.047	0.018	0.009^*^
Time of hospital stay	−0.090	0.026	0.001^*^
Motor dysfunction	−0.708	0.391	0.073

Total means total patients group; ^*^p < 0.05.

### 3.4. Data analysis of syrinx remission rate

The correlation analysis showed that age (*p* = 0.012) was negatively correlated with syrinx remission rate in the total patient group, while brainstem-related symptoms, preoperative tonsillar herniation length and different surgical methods were positively correlated with cavity remission rate (*p* < 0.05). Multiple linear regression analysis showed that age (*p* = 0.014) was a risk factor for syrinx remission, and older patients had a low syrinx remission rate. Brainstem-related symptoms were positively associated with syrinx remission rate (*p* = 0.017). Before surgery, patients who had brainstem-related symptoms had a higher syrinx remission rate.

Sex in the PFDRT group was negatively associated with syrinx remission rate (*p* = 0.025), and preoperative tonsillar herniation length was positively correlated with syrinx remission rate (*p* = 0.008). Regression analysis showed that preoperative tonsillar herniation length was the only independent factor affecting syrinx remission rate (*p* = 0.013). Patients with a longer tonsillar herniation length had a lower syrinx remission rate.

The results in the PFDD group were the same as those in the total patient group. Age was an independent risk factor for a high syrinx remission rate (*p* = 0.003). Brainstem-related symptoms were positively associated with syrinx remission rate (*p* = 0.009). The above detailed results are shown in Tables 5, 6.

**Table 5:** Syrinx remission rate bivariate correlation analysis.

**Parameters**	**Total**		**PFDRT**		**PFDD**	
	***r***	***p***	***r***	***p***	***r***	***p***
Sex	−0.055	0.458	−0.249	0.025^*^	0.079	0.433
Age	−0.186	0.012^*^	−0.111	0.325	−0.257	0.010^*^
Duration of symptoms	−0.050	0.507	−0.014	0.899	−0.139	0.164
Time of surgery	0.091	0.220	0.049	0.662	0.121	0.230
Time of hospital stay	0.03	0.686	0.084	0.455	−0.015	0.885
Blood pressure	0.013	0.858	0.146	0.198	−0.068	0.501
Paraesthesia	−0.037	0.617	0.006	0.958	−0.021	0.833
Cough headache	0.017	0.820	−0.037	0.785	0.057	0.573
Non-cough headache	0.095	0.203	0.205	0.066	0.021	0.836
Motor dysfunction	−0.065	0.385	0.039	0.733	−0.161	0.109
Brainstem-related symptoms	0.203	0.006^*^	0.201	0.072	0.213	0.032^*^
Other neurological disorders	−0.023	0.757	0.059	0.603	−0.080	0.426
Cerebellar-related symptoms	−0.110	0.138	−0.197	0.077	−0.020	0.840
Sensory deficits	0.103	0.168	−0.013	0.909	0.170	0.089
Syrinx location	0.082	0.268	0.123	0.276	0.043	0.668
The maximum anteroposterior Diameter of syrinx before surgery	0.006	0.937	−0.153	0.173	0.150	0.134
Syrinx/cord ratio before surgery	0.043	0.567	−0.088	0.432	0.147	0.143
Tonsillar herniation before surgery	0.195	0.008^*^	0.293	0.008^*^	0.110	0.275
Surgical method (PFDRT)	0.162	0.029^*^				

Total means total patients group; ^*^p < 0.05.

**Table 6:** Syrinx remission rate multiple regression analysis.

**Variable**	**Beta**	**SE**	***P*-value**
**Total**			
Constant	0.588	0.129	0.001
Age	−0.005	0.002	0.014^*^
Brainstem-related symptoms	0.357	0.149	0.017^*^
Tonsillar herniation before surgery	0.008	0.005	0.143
Surgical method (PFDRT)	0.083	0.042	0.052
**PFDRT**			
Constant	0.182	0.088	0.042
Sex	−0.121	0.075	0.108
Tonsillar herniation before surgery	0.023	0.009	0.013^*^
**PFDD**			
Constant	0.792	0.123	0.000
Age	−0.008	0.003	0.003^*^
Brainstem-related symptoms	0.510	0.192	0.009^*^

Total means total patients group; ^*^p < 0.05.

## 4. Discussion

### 4.1. Patient basic information

In present study, only the paresthesia symptoms were different between the preoperative PFDRT group and the PFDD groups (*p* = 0.032). Postoperatively, the syrinx/cord ratio in the PFDRT group was 0.35 ± 0.20, which was less than that in the PFDD group (*p* = 0.034), and the syrinx remission rate was higher than that in the PFDD group (*p* = 0.046). This suggested that patients undergoing surgery in the PFDRT group may be better relieved of syringomyelia.

Complications of the two surgical modalities have been frequently discussed. Zhao et al. (23) showed that PFDD caused complications in 21% of patients, while PFDRT caused complications in 52% of patients (23). Jia et al. (24) noted that 47% of postoperative complications were caused by PFDRT (24). Our study showed that, although the incidence of complications in the PFDRT group was higher than that in the PFDD group, there was still no significant difference (*p* > 0.05). Postoperative aseptic inflammation was the most common complication in the two groups. Subcutaneous effusion occurred in 5 (6.2%) and 5 (5.0%) patients in the PFDRT and PFDD groups. The relevant symptoms were effectively controlled after conservative treatment in the hospital. Three patients who underwent secondary surgery because of pseudomeningocele. No deaths were reported in this study. The incidence of complications in this study seems to be lower than that in most studies (16, 23–25). We suspected that an intraoperative watertight dural suture reduced the risk of CSF leakage (5, 26). Keeping the intraoperative subarachnoid space away from contaminated blood reduces CSF stimulation and reduces the incidence of postoperative CSF-related events, which is consistent with the conclusions of relevant studies (27).

### 4.2. Prognostic factors of CM-I patients with syringomyelia in the total population group

Multiple linear regression showed that motor dysfunction, cerebellar-related symptoms, and different surgical methods were independent factors influencing postoperative CCOS score (*p* < 0.05).

Surgery for type I Chiari malformation associated with syringomyelia aims at (1) restoration of normal CSF circulation at the foramen magnum, (2) reduction of the syrinx, (3) relief of the compression exerted by the cerebellar tonsils on the brain stem (27). The debate continues about whether PFDRT or PFDD has a better patient outcome. A recent survey in the United States showed that ~34% of neurosurgeons would prefer to perform cerebellar tonsillectomy after PFDD (28). In another series of 177 patients who underwent posterior fossa surgery, 135 patients (bipolar coagulation 112, subpial resection 23) who underwent tonsillar reduction showed a more pronounced reduction in the size of syringomyelia and more substantial alleviation of their symptoms. Bipolar coagulation is far easier, less risky than subpial resection. While subpial resection of the tonsils resulted in greater improvement in tonsil shape and greater enlargement of the subtonsillar spaces than did bipolar coagulation (29). Either way, Both bipolar coagulation and subpial resection aim to reduce hypohernia cerebellar tonsils and improve restricted CSF circulation in the foramen magnum, which is different from PFDD. In another observational study of 77 patients, tonsillar resection had good results in 100% of patients compared to 64.8% in those with only foramen magnum decompression (27). In contrast, other studies have not found significant differences in clinical improvement in CM-I patients with syringomyelia with different surgical procedures (16, 30–33).

In present study, different surgical methods were considered to independently influence postoperative CCOS score (*p* = 0.028), and patients treated surgically in the PFDRT group may have a better clinical prognosis than patients in the PFDD group (*p* = 0.034). We explain the impact of different surgical modalities on prognosis in terms of the origin of syringomyelia and CM-I-related symptoms. Investigators who prefer the PFDRT procedure suggested that, in CM I patients, syringomyelia may originate from the herniated cerebellar tonsil, which acts as a piston that blocks the foramen magnum in the subarachnoid space under each cardiac contraction pulse. CSF, which was propelled by piston movement, enters the spinal cord through the peripheral spinal vascular and interstitial space at each contraction cycle, forming syrinx (10). Fluids in the syringomyelia produce longitudinal oscillations with the cardiac cycle that promote syringomyelia progression (34). Motor dysfunction, paraesthesia were associated with syringomyelia in CM-I patients, and the improvement of syringomyelia may relieve clinical symptoms (27). Another study showed that 40% of CM-I symptoms are attributed to a subcerebellar tonsillar hernia (35). Batzdorf et al. (29) illustrated that headache improvement correlates strongly with reduction of the cerebellar tonsils, which can enlargement of the retrotonsillar and subtonsillar cisterns and makes contact between tonsils and the C-1 and C-2 nerve roots less likely (29). Labuda et al. (36) illustrated an interesting hypothesis that the combination of CSF restriction and reduced cervical compliance causes increased motion and strain of the cerebellum, brainstem, and upper spinal cord leading to many of the common CM-I symptoms (36).

Bao et al. (15) believed that resection of the cerebellar tonsils can resolve the cerebrospinal fluid circulation obstruction in the subarachnoid area of the foramen occipital bone and improve CSF circulation at the cranial and neck junction. Importantly, some researchers believe that cerebellar tonsillectomy does not cause neurological damage and has good clinical results (18, 37). In present study, we found that there were rhythmic up and down movements in the cerebellar tonsils during the operation. The cerebellar tonsils above the occipital foramen in patients in the PFDRT group were significantly higher than those in the PFDD group (*p* = 0.010). We speculate that cerebellar tonsillectomy increases the space of the CSF circulation, reduces the incidence of syrinx formation, and provides favorable conditions for the recovery of the syrinx. In addition, it can fundamentally solve the problems of the hernia of the cerebellar tonsil pressing the medulla, brainstem and cerebellum, providing the opportunity for recovery.

However, researchers who oppose the resection of the cerebellar tonsils believe that the risk of CSF obstruction due to arachnoid adhesion increases after tonsillectomy, which then reduces syrinx relief and aggravates clinical symptoms (25). According to Vidal et al. (38) complete arachnoid membrane is important to avoid contamination of the subarachnoid space by blood and subsequent formation of local fibrosis (38). In addition, tonsillectomy carries the risk of vascular injuries. In a relevant study, researchers pointed out that keeping the subarachnoid space away from contaminated blood during surgery can reduce the incidence of postoperative arachnoid scar formation and reduce the risk of CSF obstruction (39). Ma et al. (18) suggested that cerebellar tonsillectomy was then performed by subpial resection, which can effectively protect the surrounding structures, such as arteriae cerebelli inferior posterior (18).

Motor dysfunction and cerebellar-related symptoms were negatively correlated with postoperative CCOS scores. Dyskinesia presents as amyotrophy and myo-asthenia with a deficiency of deep reflexes, which may be caused by pressure or injury of the downward motion path (40, 41). In present study, we found that patients with motor dysfunction had less clinical improvement than patients with other symptoms, which suggested that motor dysfunction may be an risk factor for poor prognosis. Cerebellar-related symptoms caused by stretch or twist of the cerebellum as a result of a cerebellar subtonsillar hernia were risk factors for poor clinical outcome. Some studies have also shown that cerebellar-related symptoms are the least likely clinical features to be alleviated (42).

Multiple linear regression analysis showed that older patients had a lower syrinx remission rate (*p* = 0.014). Brainstem-related symptoms were positively associated with syrinx remission rate (*p* = 0.017). The central tube is the main site of syringomyelia expansion in patients with CM-I. The central tube and surrounding areas are sites of extracellular fluid transport and absorption, and mechanical or functional disturbances of these pathways can lead to central tube enlargement and interstitial edema in the surrounding area (43). With aging, the human central tube usually exhibits physiological obstruction, which causes a disordered central tube absorption mechanism, increasing the possibility of syringomyelia (44, 45). This mechanism seems to be useful in explaining the lower void remission rate in older patients. The finding that patients with brainstem-related symptoms have a high syrinx remission rate needs to be further explored. It has been reported that the greater the severity of symptoms, the greater the reduction in syrinx remission (16).

### 4.3. Prognostic factors of CM-I patients with syringomyelia in the PFDRT group

There are few relevant studies on the prognostic factors affecting the PFDRT group. Alfieir et al. (26) showed that age and duration of symptoms were significantly associated with prognosis. Patients with older age and longer duration of symptoms have a poor prognosis (26). Some studies have shown that syringomyelia diameter as well as syringomyelia features are associated with clinical outcomes (41, 46). Our analysis showed that the longer duration of symptoms, the larger preoperative syrinx diameter or the cerebellar-related symptoms would cause a lower CCOS score. We believe that patients with a long duration of symptoms have a substantially higher risk of syringomyelia and a greater degree of emptiness due to long-term CSF circulation disorders. The increased diameter of the syrinx causes more damage to the spinal cord and the bulbus medullae, which increases neurological deficits and slows postoperative recovery. Some patients may not recover from severe neurological deficits. In the PFDRT group, we found that patients with cerebellar-related symptoms may have longer duration of symptoms. These patients have prolonged stretch or twist of the cerebellum, which may cause ischemia, necrosis and permanent neurological deficits, resulting in a low CCOS score.

In addition, we found that the patients with a longer tonsillar herniation length had a lower syrinx remission rate in the PFDRT group. Some studies showed that the degree of cerebellar tonsillar hernia is not related to the extent of syringomyelia in patients. Severe tonsillar descent may be associated with a significantly lower incidence of syringomyelia (39, 42).

### 4.4. Prognostic factors of CM-I patients with syringomyelia in the PFDD group

Many studies have shown that age (26, 47), and motor dysfunction (19, 48, 49) are independent risk factors for poor prognosis in patients who undergo surgical treatment with PFDD, which were also found in our research. Multiple linear regression analysis showed that patient age and length of hospital stay were independent risk factors for low postoperative CCOS scores in the PFDD group. Patients who are older may have lower postoperative CCOS scores. Because CM-I is a congenital disease, older patients may have a longer incubation period, relatively slower progression of anatomical and clinical symptoms, and higher compliance in the posterior fossa and cranial and neck junction area (1). Older patients were slightly less sensitive to this change when they underwent surgical treatment to achieve posterior fossa decompression and restore proper CSF circulation. Therefore, elderly patients may have lower postoperative CCOS scores. This was the first study in which researchers found that a longer hospital stay was associated with a lower postoperative CCOS score. We speculate that patients with a long hospital stay may have other underlying diseases before surgery, which affects surgical and long-term clinical recovery. The independent factors influencing postoperative syrinx remission rate in the PFDD group were age and brainstem-related symptoms, which were consistent with the total population group.

### 4.5. Limitations

This study is one of the largest single-center clinical studies currently available. The present study was not grouped according to follow-up time, and the ability to explain short-term and long-term outcomes was lacking. We did not perform dynamic MRI of cerebrospinal fluid in this study. Future evaluations using cine-contrast MRI will help to show the impact of decompression surgery on CSF dynamics in the foramen magnum to assess patient outcomes. The relevant results and conclusions need to be verified in a multicenter large sample study.

## 5. Conclusion

The present study detailedly evaluated the effect of PFDRT and PFDD surgery groups through the analysis of patients' clinical outcome, imaging outcome, and predicted the relevant factors affecting the prognosis. We found that patients treated surgically in the PFDRT have a better clinical prognosis than patients in the PFDD. Preoperative motor dysfunction, cerebellar-related symptoms, and different surgical methods in CM-I patients with syringomyelia affected postoperative CCOS score. The duration of symptoms as well as the age of the patients should also be actively considered as factors affecting prognosis. Symptomatic CM-I patients with syringomyelia should undergo surgical treatment as early as possible to ensure optimal postoperative neurological recovery.

## Data availability statement

The original contributions presented in the study are included in the article/Supplementary material, further inquiries can be directed to the corresponding author.

## Ethics statement

The studies involving human participants were reviewed and approved by the Ethics Committee of the First Affiliated Hospital of Zhengzhou University. The patients/participants provided their written informed consent to participate in this study. Written informed consent was obtained from the individual(s) for the publication of any potentially identifiable images or data included in this article.

## Author contributions

MZ, YH, and DS contributed to conception and design of the study. MZ, CD, and MW organized the database. MZ and YH performed the statistical analysis. MZ wrote the first draft of the manuscript. YH, DS, FG, LZ, and SL wrote sections of the manuscript. All authors contributed to manuscript revision, read, and approved the submitted version.
