# Supplementary material for: Exploring the prognostic differences in patients of Chiari malformation type I with syringomyelia undergoing different surgical methods
Source: Front Neurol. 2023 Jan 4;13:1062239. doi: 10.3389/fneur.2022.1062239 (PMC9846178; doi:10.3389/fneur.2022.1062239)
Supplement: Supplementary file 1 [file Table_1.DOCX]

**Supplementary**

| Feaature | Total | PFDRT（n=81） | PFDD（n=101） | P Value |
| --- | --- | --- | --- | --- |
| Paraesthesia | 80（86.96%） | 43（89.58%） | 37（84.09%） | 0.435 |
| Cough headache | 58（93.55%） | 27（96.43%） | 31（91.18%） | 0.620 |
| Noncough headache | 12（80.00%） | 8（88.89%） | 4（66.67%） | 0.525 |
| Motor dysfunction | 35（66.03%） | 15（71.43%） | 20（62.50%） | 0.502 |
| Brainstem-related symptoms | 3（75.00%） | 2（100.00%） | 1（50.00%） | 1.000 |
| Other neurological disorders | 6（85.71%） | 3（100.00%） | 3（75.00%） | 1.000 |
| Cerebellar-related symptoms | 10（83.33%） | 6（85.71%） | 4（80.00%） | 1.000 |
| Sensory deficits | 22（75.86%） | 8（80.00%） | 14（73.68%） | 1.000 |

Table 1 Patient’s recovery of clinical symptoms at follow-up
